# The Transboundary Crisis: Why we are unprepared and the road ahead

**DOI:** 10.1111/1468-5973.12241

**Published:** 2018-07-22

**Authors:** Arjen Boin

**Affiliations:** ^1^ Department of Political Science Leiden University Leiden The Netherlands

## Abstract

Modern societies rely on complex technological systems that are deeply intertwined with other complex systems that stretch across geographical, judicial and administrative borders. When threats emanate from this transboundary space, national governments are often surprised and discover that existing crisis management arrangements do not suffice. This article describes the political and administrative challenges that accompany transboundary crises. It argues that arrangements and processes that work reasonably well for “bounded” crises are unlikely to work in the case of transboundary crises. It formulates an agenda for political debate and academic research. The bottom line is that we need to rethink traditional crisis management arrangements in order to prepare for these increasingly common type of threats.

## 1 THE IMPOSSIBLE CRISIS

Much has been done in recent decades to prepare modern society for all sorts of crises and disasters. One might say that many societies have never been as prepared for crises and disasters as they are today. That is a good thing. But here is the bad news: Modern societies are woefully underprepared for dealing with a type of threat that is on the rise. This is what I refer to as the Transboundary Crisis.

The Transboundary Crisis effortlessly exceeds geographical, policy, cultural, public–private and legal boundaries that normally enable public managers to classify, contain and manage a crisis. It escalates rapidly and mutates constantly, creating confusion about causes and possible consequences. It ends up on many administrative tables, but it is not obvious which of those tables is or should be “in the lead.” These features make a fast and adequate response difficult, to say the least.

The Transboundary Crisis comes in many guises:

- In 2002, a mysterious virus spread from China, via Hong Kong, to 37 countries causing hundreds of deaths. The SARS virus caused a major health crisis in Toronto.
- In 2008, the American investment bank Bear Stearns declared bankruptcy, after which the U.S. economy collapsed together with a set of European economies (Portugal, Ireland, Spain and Greece).
- In 2010, a volcano on Iceland (Eyjafjallajökull) paralysed air traffic across the world. Tens of thousands of travellers got stuck at airports everywhere.
- In 2015, hundreds of thousands of immigrants entered Europe from Turkey and marched from Greece to Sweden. The immigrant flow caused political and humanitarian crises along the routes.

In all these examples, national governments were confronted with a crisis that had origins in faraway domains. However well prepared they may have been for traditional crises, they soon discovered that their response repertoire was insufficient in the face of the transboundary challenges.

When the State has no answer to a crisis, consequences follow. In times of crisis, the public expects representatives of the State to take charge. It is, after all, a core task of the State to protect its citizens against the consequences of threat and calamity. If the State fails in this core task, the legitimacy of public institutions and the reputation of political leaders are undermined. That is problematic, especially when the legitimacy of public institutions is already under question.

The Transboundary Crisis is the ultimate nightmare for crisis managers. It marks the moment they discover their traditional crisis arrangements do not suffice in the light of the political–administrative challenges that this crisis brings. It sheds light on a structural governance deficit, which presents politicians with a pressing design, let alone management, challenge.

This study offers a roadmap for a discussion about possible solutions. This roadmap hinges on a strategic choice between two options that emerge from our discussion of theory: move backward by decoupling from modern systems or move forward by strengthening transboundary crisis management capacities.

## 2 THE TRANSBOUNDARY CRISIS: CHARACTERISTICS

Transboundary crises may come in different guises, but they share common characteristics that make them difficult to manage:

1. *Multiple domains, multiple manifestations:* The Transboundary Crisis reaches across multiple countries and/or multiple policy areas. There is no defined geographical location (a “Ground Zero”) or policy sector around which to organize. That creates diversity in perspective: What in one domain is experienced as a problem of scarcity may become a matter of public safety in another; what in one country is considered a local matter is *Chefsache* in another country.
2. *Incubation and rapid escalation:* The Transboundary Crisis is characterized by periods of slow, often imperceptible development and phases of rapid escalation. Europe's immigration crisis, for instance, attracted little attention for years. The escalation came seemingly out of nowhere when thousands of people drowned in the Mediterranean, and refugee flows reached the coasts of Greek and Italian islands.
3. *Hard to chart:* The root causes of a crisis that originated in another country or sector are difficult to comprehend. Causes are unclear, possible consequences seem uncertain, and escalation is unpredictable. When it is all over and the report on the crisis is written, it may become clear what happened (and how it could have been avoided). But *during* the crisis, it is extremely difficult to establish the most basic developments. The Transboundary Crisis brings rude surprises.
4. *Multiple actors, conflicting responsibilities:* The Transboundary Crisis does not fall neatly into a defined domain, with a clear division of tasks and responsibilities. The Transboundary Crisis challenges multiple actors with various responsibilities. It is not clear which actor is responsible for what or who has the capacities to perform certain tasks. The Transboundary Crisis blurs the organizational boundaries that normally facilitate an effective response.
5. *No ready‐made solutions:* For most policy issues, possible solutions exist. They may be controversial or taken for granted. But it is usually clear what the discussion is or should be about. The Transboundary Crisis defies easy or conventional solutions. And what works here may not work there. Consider the cyber domain: It is rarely clear who or what is behind a cyber disruption; it is often even less clear who can do what to protect a critical system against a cyber attack.

## 3 THE RISE OF THE TRANSBOUNDARY CRISIS: CORE CHALLENGES

The Transboundary Crisis brings a critical challenge to any administrative system that is based on boundaries and demarcation. By crossing borders, the Transboundary Crisis challenges borders. In a democratic state that is based on the principle of political accountability and makes use of the bureaucratic organization form, most organizations are organized around demarcated areas of expertise and authority. The bureaucratic organization is based on boundaries among task fields, responsibilities, divisions, departments and policy sectors.

Two mechanisms have been traditionally used to address blurring of borders: coordination (negotiating boundaries) and centralization (transcending boundaries). These mechanisms can be problematic in the best of times; they are especially problematic in a transboundary crisis.

*Coordination* mechanisms may work fine for complex problems and the traditional crisis. The mechanisms, however, do not work in the world of the Transboundary Crisis for two reasons. First, in a transboundary crisis, it is not clear who the critical actors are or should be, and what their authority in the matter is. Second, it is hard to establish or negotiate ownership in a short time frame (time is always scarce in a crisis). We may thus say that the Transboundary Crisis robs bureaucracy of its most effective tool.

*Centralizing emergency powers* in the hands of a leader or a central body is the traditional catch‐all solution. In the Roman Empire, unlimited powers were placed in the hands of a dictator. In modern democracy, crisis centralization is still a valued mechanism. But it comes with constraints: It is not easy to centralize power, and it does not happen often. Moreover, the “high command” is not always defined clearly enough, and the mechanisms that should regulate such a concentration of power are cumbersome. A quick look at the legal terms that condition the authorization of exceptional violence (the deployment of special police units) makes clear that a tension exists between legal considerations and the required speed of action. This tension can often only be circumvented in an environment in which political actors know and trust each other.

In a Transboundary Crisis, the nation state may not be the only actor. But how to centralize power in an international context? Governments are reluctant to shift decision‐making authority to international institutions. Think, for example, of EU agencies, which in principle could play a decisive role during a transboundary crisis. But these organizations were never endowed with decision‐making powers. We should, then, not be surprised that an EU agency such as FRONTEX accomplished so little during the immigration crisis that peaked in 2015.

In sum, transboundary crises pose a wide and deep challenge to the standing governance arrangements of democratic states. That is problematic. The state is left rudderless in a time when citizens look to their elected leaders and trusted institutions to navigate them through the storm. A transboundary crisis can thus rapidly become a crisis of legitimacy.

A vicious cycle threatens. The effectiveness of the crisis response relies to a large extent on legitimacy. But the legitimacy of public institutions is already under attack. If institutions do not function effectively during a crisis, they lose even more legitimacy. The Transboundary Crisis makes vulnerable institutions even more vulnerable.

## 4 PREPARING FOR THE TRANSBOUNDARY CRISIS: TWO OPTIONS

What can be done to protect our country, our prosperity and our well‐being in a world of new, unprecedented crises that effortlessly bypass existing lines of defence? To answer this question, we must understand the underlying drivers of the Transboundary Crisis.

Two books, both classics, provide a great starting point: Barry Turner's (1978) *Man‐made Disasters* and Charles Perrow's (1984) *Normal Accidents*. Both authors focus on the relentless modernization of socio‐technical systems. We build increasingly complex systems that we connect to other complex systems with one goal: to enhance the efficiency of critical processes (such as food supply, transport, production chains, Internet and energy), that is, to increase the speed of service delivery at ever‐lower costs. The thesis that emerges from both books can be summarized as follows: These “highways of efficiency” become the “highways of failure” that allow, if not actually enable, routine disruptions to travel very quickly from one system to another.

Perrow's argument is simple and convincing. If a system becomes evermore complex, it also becomes evermore difficult to understand. When something goes wrong, it is not immediately clear for system operators what is happening. If a complex system is tightly connected with other systems, we know that even a small disturbance can affect the functioning of these other systems. By the time the problem is recognized in one system, others may have been infected.

Complexity and tight coupling create fertile ground for small incidents to jump from one domain to another and thus escalate into larger‐scale crises. New threats exploit these “highways of failure.” The rise of revolutionary technologies—artificial intelligence, DNA editing, drones, 3D‐printing, self‐propelled cars, the “internet of things”—brings happiness and economic prosperity, but they also speed up failure and create unknown shortcuts for unprecedented threats.

From this theoretical perspective, two options emerge. A country can move backward by *decoupling* from the modern complexity ecology or move forward by a strategy of *protecting complexity*.

### 4.1 Moving backwards: building walls

Perrow opted for the way back: simplify and unbundle critical systems. If complexity and tight coupling are the problem, Perrow's solution—decoupling—is the obvious solution: return to simpler systems that are isolated from other systems. By decoupling from modernity, many crises simply cannot happen.

Insulation and retrenchment: These are solutions of all times. In the political domain, we recognize that solution in the romanticizing of past times and the call for new cultural, geographical and administrative boundaries. The leaders of Hungary and the United States want to build walls; the United Kingdom wants to draw up the bridge to the European Union. The movements are based on the promise that we can shield citizens from the adverse effects of modernity.

In professional domains, this is also a common solution. The shutdown (*islanding)* of ICT systems protects against viruses and hacks. Vaccinations stop outbreaks. The storm diagrams of national railways, the scarcity priority lists of the electricity company or the gates, canals and stewards in the football stadium: professionals routinely create boundaries to stop the escalation of crises.

The choice for decoupling does not come free, however. Withdrawal entails a decoupling from the benefits that complex systems generate. It is a costly affair, as economists are fond to explain. But this rational argumentation collides with the growing unease about the negative effects of modern systems. In times of uncertainty, the call for entrenchment behind hard borders may not be evidence based, but it is intuitively convincing and politically attractive. We should therefore expect that “decoupling” remains an option as long as modern systems generate uncontrollable hazards.

### 4.2 Moving forward: Preparing for an effective response

An alternative (or complimentary) strategy is prepared in the face of complexity. If we accept that disturbances can and will emerge, perhaps we are better off investing in early detection and timely intervention. Aaron Wildavsky (1988) argued that enhanced *resilience* through a *trial‐and‐error* strategy, relying on the human capacity for innovation and learning, is a much more fruitful strategy than a short‐sighted focus on prevention through entrenchment.

Resilience became a dominant feature of many policy efforts in the domain of safety and security. Governments that are unable or unwilling to protect their citizens against systemic risks appeal to the ability of citizens (also organizations and communities) to “absorb shocks” and “bounce back” after a disruptive event. The underlying idea is that citizens know what is good for them. In this line of thinking, it is not necessary that government arranges full protection for all citizens from all those complex systems surrounding them.

It is true that citizens in the first hours after a disaster usually are typically able to organize themselves and help each other. But this is not necessarily true for the longer term. The people who remained behind in New Orleans when Hurricane Katrina destroyed the city were the poor, the elderly and the sick. They did not have the means to bounce back, let alone move forward. Seen from this perspective, resilience is a dubious strategy. It leaves citizens to their fate, just when they most need the State.

Any false promise of resilience eventually leads us back to Perrow's decoupling strategy. Letting citizens fend for themselves when under mortal threat touches a core task of the State. If the State turns its back on its citizen—the same State that never fails to emphasize the blessings of interconnected systems—the “way back” will inevitably come to look attractive. If the State cannot protect us, perhaps we *should* build that wall—or so the argument goes.

Resilience is an attractive but politically risky strategy. But what is the alternative? Can we really manage the risks that modern systems produce? To answer this question, let us briefly explore what would be required, and whether it is possible, to create response capacity for the Transboundary Crisis. We look at three major tasks that are crucial for any major crisis response: detect emerging threats, understand how they unfold and run a coordinated response while preserving public support (cf. Boin, ‘t Hart, Stern, & Sundelius, 2016). Let us take a quick look at these three tasks.

#### 4.2.1 Detection of vulnerabilities

Given their magnitude, it is essential to detect any Transboundary Crisis as soon as possible. Only if escalation across boundaries is recognized in time, can intervention efforts stand a chance. Detection begins with understanding the many ways in which incidents can develop into cross‐border threats. How are systems connected? How can a system be infected by a disruption in another system? How can one system be protected against another without undermining the fruits of the existing connection? We need to understand the vulnerabilities of complex and tightly coupled systems.

Vulnerability thinking is still in its infancy. Intelligence services seek to recognize potential threats in a timely manner. Hedge funds bet on “big data” to foresee political upheavals. The EU has more than 100 detection and early warning mechanisms in place (but nobody knows if they are effective). Research funded by the EU (Horizon 2020) focuses on understanding escalation mechanisms in critical infrastructures. Complexity researchers study “tipping points.” Other researchers focus on the ability of people in control rooms to detect early aberrations in critical processes. The need for detection mechanisms is clearly and widely acknowledged, but we await major breakthroughs. The Transboundary Crisis will continue to surprise us for the time being.

#### 4.2.2 Transboundary sense‐making

The Transboundary Crisis is difficult to comprehend. The causes are hidden in system complexity and pile up when the dominoes start falling. To understand how a threat unfolds, where exactly and how quickly, it is necessary to bring together as much relevant information as possible, that is, to authorize, analyse and share it with the right parties—quickly and effectively.

Babylonian confusion is then almost guaranteed. Many actors are involved. It often is not clear where information should come together. It is usually not even clear what information exists, especially as actors appear and disappear. Critical information is hidden in technical concepts, clashing paradigms and different languages. Moreover, critical information gets “stuck” in unexpected places.

Standard solutions consist of investments in procedures, specialists and technical equipment (crisis centres now have wall‐to‐wall screens). But those solutions cannot address a key weakness: the inability of information specialists to translate operational information into strategic information.

The U.S. Homeland Security Operations Center, opened with much fanfare in 2004, provides a striking example (Boin, Brown, & Richardson, 2019). It had a huge budget, hundreds of specialists and a clear task: collect all information during a crisis and generate an accurate picture of the situation. This same centre was the sink hole where critical information about the broken levees in New Orleans disappeared. It took the federal authorities more than 24 hours to understand that the city was under water.

The solution does not lie in the development of new technologies. What is needed is an approach that helps information managers to quickly collect information from a variety of organizational domains. They must learn to locate sources of critical information; they must also learn to make sense of that information, which is likely to be difficult as the information emanates from very different sources.

#### 4.2.3 The establishment of transboundary decision‐making powers

It is a truism that critical decisions must often be made quickly on the basis of very little information when in a crisis. From a legitimacy perspective (see below), it is important that those critical decisions are made by the appropriate officials or institutions.

The Transboundary Crisis makes this quite tricky, as it challenges the underlying logic of bureaucracy (where responsibility is tied to a person, position or institution). When a crisis involves multiple actors, each with their own responsibilities, interests and working methods, it must be clear who is authorized to decide what. This rarely has been determined beforehand, as a transboundary crisis tends to involve a unique constellation of actors.

As we have seen, the typical solution to this type of problem involves a combination of centralization and coordination. Centralization is hard enough for the more routine crises; it is even less likely to work in the international arena. National governments do not lightly cede authority to international organizations (certainly not during a crisis where so much is at stake). The international arena has produced some innovative solutions: think of NATO's article five (centralization in case of defined threats) and the European Central Bank (centralization by stealth).

The most important problem is that crisis centralization tends to create serious legitimacy issues. If the ownership on critical issues is allocated to an official or institution, and thus *not* allocated somewhere else or taken away from someone, it is crucial that the “crisis owner” can rely on political and public support. If this is not the case, the legitimacy of public institutions may come under pressure. Without a solid legitimacy basis, effective crisis management becomes difficult.

Centralization that has not been subjected to processes of democratic deliberation and control is risky in this regard. The European Central Bank became increasingly powerful during the financial crisis and is hardly subject to democratic control. The impotence of the European Parliament is telling. The European Central Bank has made decisions that large groups of citizens perceive as unjust. The lack of democratic control opens the door for politicization of the crisis response.

Coordination, on the other hand, may work. But that requires a degree of “instant trust” that is often lacking between actors that have never worked together before. Promising initiatives can be found at the interface of public and private. For instance, the Netherlands has the ICT Response Board in which private parties work with government to prepare for cyber disasters. The Louisiana Business Emergency Operations Center is another innovative example. The question is whether such practices can be scaled to the international level.

Theoretical progress is being made. By formulating transboundary crisis management as a *collective action problem*, we can apply theoretical insights from this body of research. This type of research may provide a view of the conditions under which crisis cooperation is possible, and the strategies that may be helpful. One such strategy, for example, is based on that idea of “instant trust”—unilaterally taking the first step, without being sure of reciprocity (Majchrzak, Jarvenpaa, & Hollingshead, 2007).

But, overall, we can state that mechanisms for the delineation of authority are lacking for transboundary crises. In the absence of serious political discussion, real progress will likely only happen after a disastrous encounter with a transboundary crisis.

## 5 HOW TO PROCEED?

The Transboundary Crisis is, of course, not a new phenomenon. Crises have always traversed boundaries. Think of the plague sweeping across Europe, the 1918 flu epidemic, food shortages in the Roman Empire or the two world wars. The optimist may claim that the Western world has managed the really big threats in the postwar era. The pessimist may argue that the connections among international systems have spectacularly increased, problems have become more complex, and the number of actors has increased dramatically. Even if the pessimist is only half right, we may justifiably ask whether we are sufficiently prepared.

The good news is that crisis management has professionalized in the past few decades. Since the beginning of this century (after the Millennium computer bug and 9/11), western countries have heavily invested in crisis management capacity, both in the public and private sectors. Municipalities, schools, hospitals, businesses—crisis management is now firmly on the radar just about everywhere. More, even though public bureaucracies are not designed to deal with exceptional situations, “work arounds” have been created to deal with routine crises.

I have argued, however, that these existing crisis management structures are no match for the Transboundary Crisis. When the system under threat becomes spread too far, and workarounds do not emerge in time, the results may be catastrophic. SARS, the financial crisis, Hurricane Katrina—we know what can happen.

The Transboundary Crisis requires a different way of organizing and working. The question, then, is: who can shape the new ways of organizing and responding? There are several possibilities (which are not mutually exclusive).

1. We leave it to the *national crisis institutions*. We can instruct these institutions to pay more attention to the transboundary dimensions of crises.

It will not be easy to introduce a new way of working in institutions that are built around traditional practices that work for traditional incidents and crises. These organizations do not have to unlearn standing practices; they must learn to deal with the Transboundary Crisis. We must, in other words, make those institutions “ambidextrous.” This will require a lot of work, because national crisis institutions have little affinity with transnational and cross‐border crisis management.

- *Invest in existing international organizations*. Perhaps it is better to invest in organizations that are already active in cross‐border domains. NATO and the European Union have in recent years developed crisis management capabilities which, in principle, are intended for cross‐border crises.

The EU, in particular, has developed in embryonic form abilities needed for a Transboundary Crisis. But these capabilities and assets are scattered across the many agencies and Commission parts of the EU. And the crisis performance of the EU is not always considered effective or legitimate. In particular, the response to the financial crisis and the immigration crisis has become sources of controversy. The EU will need time to become an effective and legitimate actor in the crisis domain.^1^

- Build *transboundary crisis management institutions*. Think in terms of a new organization, with new people, a respected leadership, real powers and a realistic budget. An Institute that invents and tests new forms of crisis management practices. New processes and forms of organization that can effectively address the Transboundary Crisis.

The advantage is that a brand new institution can devise novel methods or ways of thinking from scratch. That is, at the same time, the downside: It will take a long time and the outcome is by no means guaranteed.

We conclude by setting out a research challenge. It is important to study the different guises in which the Transboundary Crisis comes, which allow for classification. It may well turn out that some types of Transboundary Crises are more amenable to certain interventions (such as centralization and coordination, decoupling or resilience) than others. It may well be that certain types are more politically or analytically challenging. The research challenge is to find out how characteristics relate to preparatory and management efforts. We may discover that certain types are intractable, whereas others may lend themselves to early detection and intervention. By studying cases of actual crises and near misses, we should be able to enhance our understanding of the Transboundary Crisis.

## Endnotes

1 For a recent research project, see the results of the EU‐funded Transcrisis project: http://www.transcrisis.eu.
